# Promptness of oxytocin administration for first-line treatment of postpartum hemorrhage: a national vignette-based study among midwives

**DOI:** 10.1186/s12884-022-04648-5

**Published:** 2022-04-23

**Authors:** S. Voillequin, P. Rozenberg, Ph. Ravaud, A. Rousseau

**Affiliations:** 1 Department of Obstetrics and Gynecology, Strasbourg University Hospital, Strasbourg, France; 2 INSERM UMR1018 “Clinical Epidemiology Team”, Research Center on Epidemiology and Population Health (CESP), UVSQ, Paris Saclay University, Villejuif, France; 3 Midwifery Department, Strasbourg University, Strasbourg, France; 4 Department of Obstetrics and Gynecology, Poissy-Saint Germain Hospital, Poissy, France; 5 INSERM UMR1153, Centre of Research Epidemiology and Statistics (CRESS), Université de Paris, Paris, France; 6 Center for Clinical Epidemiology, Assistance Publique des Hôpitaux de Paris (AP-HP), Hôpital Hôtel Dieu, Paris, France

**Keywords:** Oxytocin administration, Treatment guidelines, Midwives, Case-vignette, First-line treatment

## Abstract

**Background:**

Postpartum hemorrhage (PPH) remains a leading cause of maternal morbidity and mortality worldwide. Midwives play a key role in the initial management of PPH. Uterotonic agents are widely used in its prevention and treatment, with oxytocin the first-line agent. Nonetheless, a standardized guideline for optimal dose and rate of administration has not been clearly defined. The aim of this study was to investigate French midwives’ practices regarding first-line oxytocin treatment and the factors influencing its delayed administration.

**Methods:**

This multicenter study was based on clinical vignettes of PPH management collected using an anonymous online questionnaire. A random sample of midwives from 145 maternity units in France from 15 randomly selected perinatal networks were invited to participate by email. The Previously validated case vignettes described two different scenarios of severe PPH. Vignette 1 described a typical immediate, severe PPH, and vignette 2 a less typical case of severe but gradual PPH They were constructed in three successive steps and included multiple-choice questions proposing several types of clinical practice options at each stage. For each vignette separately, we analyzed the lack of prompt oxytocin administration and the factors contributing to them, that is, characteristics of the midwives and organizational features of maternity units. Bivariate analysis and multivariable logistic regression analysis were applied.

**Results:**

In all, 450 midwives from 87 maternity units provided complete responses. Lack of promptness was observed in 21.6% of responses (*N* = 97) in Vignette 1 and in 13.8% (*N* = 62) in Vignette 2 (*p* < .05). After multivariate analysis, the risk of delay was lower among with midwives working in university maternity hospitals (ORa 0.47, 95% 0.21, 0.97) and in units with 1500 to 2500 births per year (ORa 0.49, 95% CI 0.26, 0.90) for Vignette 1. We also noticed that delay increased with the midwives’ years of experience (per 10-year period) (ORa 1.30, 95% CI 1.01, 1.69).

**Conclusions:**

This study using clinical vignettes showed delays in oxytocin administration for first-line treatment of PPH. Because delay in treatment is a major cause of preventable maternal morbidity in PPH, these findings suggest that continuing training of midwives should be considered, especially in small maternity units.

**Supplementary Information:**

The online version contains supplementary material available at 10.1186/s12884-022-04648-5.

## Background

Postpartum hemorrhage (PPH) remains a leading obstetric emergency, causing 25% of maternal deaths worldwide and accounting for up to 75% of severe maternal morbidity [1]. Most of these deaths could be avoided through the use of prophylactic uterotonics during the third stage of labor and by timely and appropriate management [2–5]. Confidential enquiries of maternal deaths in several countries have identified deficiencies in care, compared to guidelines, in 90% of cases [2, 3, 6–8]. A systematic review of rigorous evaluations concluded that explicit guidelines improve clinical practices, when introduced in a context of such assessments [9]. Confidential enquiries into maternal deaths in the United Kingdom have demonstrated that wide variations in management lead to higher levels of morbidity [3].

The World Health Organization (WHO) recommends the use of uterotonics to prevent PPH during the third stage of labor for all births. Oxytocin given prophylactically in the third stage of labor is effective in reducing PPH and is therefore considered a component of active management of the third stage of labor [10, 11]. For this reason, prophylactic oxytocin injection is routinely recommended internationally [12–15] and is part of the French guidelines [16]. This prevention aims to prevent the uterine atony that is responsible for 70–80% of PPHs and remains their single most common cause [17].

Nonetheless, guidelines about PPH management are not consistent or standardized. Uterotonic drugs are the primary treatment when prevention fails and excessive bleeding occurs. Based on moderate quality evidence [18–20], the WHO recommends oxytocin as the first-line uterotonic drug for PPH treatment, including for women who have already received it for PPH prophylaxis. Sometimes however, guidelines prescribe a two-stage administration, alone or combined with another uterotonic if necessary, and sometimes without a defined temporal sequence of administration [12–16, 20]. These recommendations, which also vary for doses, routes of administration, and regimens for the administration of oxytocin treatment, are mainly based on expert consensus [12–16, 21]. All guidelines agree, however, that oxytocin should be used prophylactically during the third stage of labor and therapeutically immediately after PPH is diagnosed [12–16], for the timing of its administration can affect the volume of maternal blood loss [22–24]. A French population-based cohort study confirmed that the risk of morbidity rises when initial care is delayed. In particular, the delay of oxytocin administration was associated with higher rates of severe PPH [25]. In addition, several studies treat prompt oxytocin administration (< 15 min) as a criterion of quality care [6, 7, 19, 25–27].

The French national guidelines state that if PPH occurs, the first obstetric procedure to be performed is the manual removal of the placenta or, if already delivered, manual uterine exploration (for clot removal). This procedure should be followed by an injection of 5 to 10 IU of oxytocin even if the prophylactic injection was performed. Persistent bleeding within 15 to 30 min after diagnosis and initial management of PPH should lead to the implementation of second-line management. These guidelines are applicable nationwide, and initial management does not differ according to maternity unit [21].

Members of our team have previously reported deviations from guidelines especially at the individual level and for oxytocin use specifically through case-vignettes [28, 29]. This study was specifically designed to investigate the promptness of oxytocin administration for first-line PPH treatment. Most previous studies have focused on oxytocin administration as a preventive measure [30–35]. A first step in resolving guideline implementation issues to improve care is identifying the specific deviations from the optimal care described in guidelines and their determinants.

In France, midwives provide the initial management of nearly all cases of PPH, as they await the rest of the anesthetic and obstetric team. They hold a master’s degree in medical training, and the profession of midwife is registered in the public health code as a medical profession, just as the professions of doctors and dentists are. They can therefore prescribe some drugs, including oxytocin. The combination of these factors is why we chose to study them specifically.

Our primary objective was assess the variations in the promptness of oxytocin administration for first-line PPH treatment. Our secondary objective was to identify factors potentially associated with delayed oxytocin administration among midwives’ characteristics and maternity units’ organizational features.

## Methods

This multicenter cross-sectional study took place from January to April 2014. Midwives were requested to respond to an online survey, in which they answered multiple-choice questions about how they would manage two case-vignettes of PPH and a short questionnaire.

### Survey instrument: case-vignettes

Le plan, les détails et les résultats de l'étude ont déjà été publiés [28, 36].

Les vignettes cliniques sont des instruments de mesure de la qualité de la pratique et peuvent être utilisées pour évaluer les pratiques professionnelles [36–38]. Les vignettes ont été élaborées et validées en comparant les pratiques réelles avec les réponses aux vignettes dans une étude de validation précédente. Ces deux vignettes de cas dynamiques ont été choisies par trois sages-femmes et trois obstétriciens parmi 66 vignettes précédemment validées parce qu'elles correspondent à deux situations fréquentes différentes [28]. Ils couvraient trois étapes représentant les mesures que les lignes directrices dictent devraient être prises toutes les 15 minutes [18].

Vignette 1 described a typical immediate and severe PPH, involving heavy bleeding and consequent blood loss estimated at 800 ml, 10 min after delivery, with the placenta not delivered.

Vignette 2 was a less typical case of severe but gradual PPH with a constant trickle of blood after placental delivery. Vignette 2 showed PPH with minimal persistent bleeding and an estimated 650 ml of blood loss, 1 h after delivery. Bleeding and maternal condition were illustrated by photographs (see the Additional files 1 and 2). Both vignettes mention that active management of the third stage of labor was performed; it includes the prophylactic injection of oxytocin at the time of delivery.

For each vignette and for each step (15-min), midwives were asked to answer multiple-choice questions regarding its pharmacological and non-pharmacological management as well as actions related to communication-monitoring-investigation. The drug treatments offered as choices were: an antibiotic, oxytocin, misoprostol (a prostaglandin E1 analog), methylergometrine, sulprostone (a prostaglandin E2 analog), and tranexamic acid. The choices for oxytocin management in each step concerned its administration (yes/no), and when yes, its route and dose. Midwives could not return to a previous step to change their answers.

### Survey administration

We invited 15 French regional perinatal networks, including 212 maternity units, to participate in the study. French perinatal networks include all level 1, 2, and 3 public and private maternity units in their region. Two networks, accounting for 34 maternity units, refused to participate, while 33 maternity units had closed, refused, or were then without supervisors. We were thus able to send follow-up invitations to participate to 13 networks and 145 maternity units.

An email was sent to the supervising midwife of each unit, explaining the purpose of the survey and including a link to the survey website (55 of these units never responded to this follow-up invitation). This email was forwarded to all midwives who worked during an arbitrarily chosen period in the unit’s delivery room (Monday through Sunday, January 13–19, 2014). Two email reminders were sent at two-week intervals to the midwives through their supervisors.

### Main outcome

The binary primary outcome was defined as non-adherence to guidelines. The criteria for assessing responses to the vignettes were determined by the same committee of experts that drafted the guidelines (Table 1) [28]. Practice was not considered prompt, that is, adherent, unless oxytocin was administered in step 1 (< 15 min) in both vignettes. A delay of 15 min or longer cannot be considered good-quality management [6, 7, 19, 25–27]. The error was evaluated separately for each vignette.

**Table 1:** Criteria for evaluation of adherence to guidelines

**Pharmacological management**	
First line uterotonic: oxytocin in step1	
Second line uterotonic: sulprostone (prostaglandin E2 analogue) in step2	
No misoprostol (prostaglandin E1 analogue) in any step	
**Non-pharmacological management**	
Manual placental delivery, manual examination of the uterine cavity in step1	
No intrauterine tamponade in step 1	
No torsion of the cervix in step 1	
Uterine massage in steps 1 or 2	
Cervical examination with speculum in steps 1 or 2	
No surgical treatment in steps 1 or 2	
No selective arterial embolization in steps 1or 2	
Surgical treatment, selective arterial embolization and/or intrauterine tamponade in step 3	
**Communication, monitoring and investigation**:	
Alert other members of the team in steps 1 or 2	
Venipuncture with blood count, hemostasis in steps 1 or 2	
Resuscitation measure in steps 1 or 2	

### Study variables

A self-administered questionnaire provided data on the midwives’ individual characteristics and the organizational features of their workplaces.

The following characteristics were collected for midwives: gender, age in years, experience (years of practice), and whether they worked full-time (yes or no). The organizational characteristics of the units included: status (non-university or university); level of care, categorized as 1, 2 or 3 (Level 1: no neonatology department; Level 2: presence of a department of neonatology and special care in the same building or immediate proximity to the site of delivery; and Level 3: neonatal intensive care present in the same building); and volume of births per year, categorized as < 1500, 1500–2500, > 2500.

### Statistical analysis

Categorical variables were described with numbers and percentages. Proportions were compared with the Chi-2 test or Fisher’s exact test, as appropriate. Numerical variables were described by their means, standard deviations (SD), and medians, interquartile range (IQR). They were compared with Student’s test or Wilcoxon’s test, as appropriate.

We used logistic regression models to investigate the relations between prompt oxytocin administration and potential risk factors (characteristics of the midwife and maternity unit). Reference categories were determined from the literature. The binary logistic regression was then adjusted to include only those potential variables significant at *p ≤* .20 in the multivariable model. Multicollinearity was assessed by computing the variance inflation factor (VIF); because it was not found to be present, all identified variables were included in the regression models. Results were expressed as odds ratios (OR), with their 95% confidence intervals (CI).

We investigated the effects of variations in the intracluster correlation co-efficient (ICC). As the ICCs for the maternity units were low (ICC = 0.13) as were those for the perinatal networks (ICC = 0.12), we considered that it was not necessary to take the center effect into account with a multilevel model and therefore performed logistic regression.

All *p*-values are two-sided and considered statistically significant if less than .05. Statistical analysis was conducted with R statistical software, version 1.4.1021.

## Results

### Study population

**Fig. 1:**
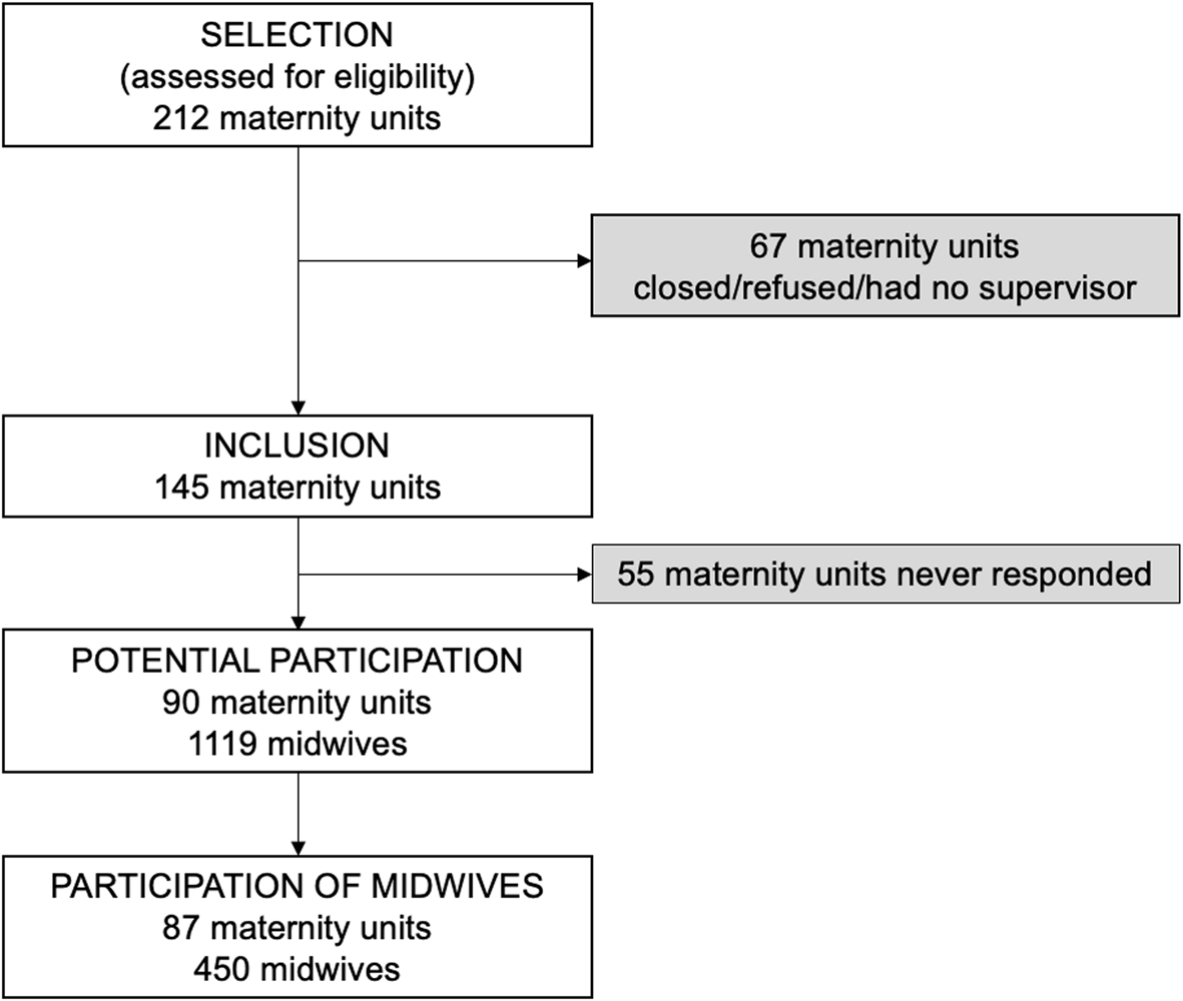
Flowchart

We obtained complete responses from 450 midwives from 87 maternity units (Fig. 1). The figure below presents the study flowchart.

Table 2 reports the midwives’ characteristics. Their mean age was 34.72 years (+/− 8.44) and 94.4% were women. Most midwives had a full-time job (74%) and worked in non-university maternity units (77.1%), level of care 2 (43.8%), and with fewer than 1500 births per year (40.4%).

**Table 2:** Midwives’ characteristics (*N* = 450)

Characteristics	*N* = 450
***Individual characteristics***	
Gender, n (%)	
Men	25 (5.6)
Women	425 (94.4)
Age, year, mean (SD), median (IQR)	34.72 (8.44), 33 (28–40)
Experience, year, mean (SD), median (IQR)	11.38 (8.71), 7 (4–17)
Full-time job, n (%)	333 (74.0)
***Organizational characteristics***	
Maternity unit status, n (%)	
Non-university	348 (77.3)
University	102 (22.7)
Level of care, n (%)	
Level 1	128 (28.4)
Level 2	197 (43.8)
Level 3	125 (27.8)
Volume of births per year, n (%)	
< 1500	182 (40.4)
1500–2499	121 (26.9)
> 2500	147 (32.7)

### Description of variation in the prompt administration of oxytocin

The promptness of oxytocin administration is described in Table 3. Administration was prompt more often in Vignette 2. Oxytocin was not administered in step 1 in 21.6% (*n* = 97) of the responses to Vignette 1 and in 13.8% (*n* = 62) for Vignette 2 (*p* < .01). A statistically significant difference in administration between the vignettes was observed at each step. Oxytocin was never used in any step in 12.9% (*n* = 58) of responses for Vignette 1 and 7.8% (*n* = 35) for Vignette 2 (*p* = .01).

**Table 3:** Promptness of oxytocin administration by step between Vignette 1 and Vignette 2 (*N* = 450)

	Vignettes		***p-value***
	V1, *N* = 450	V2, *N* = 450	
**Oxytocin step 1 (< 15 min), n (%)**			**<.01**
no	97 (21.6)	62 (13.8)	
yes	353 (78.4)	388 (86.2)	
**Oxytocin step 2 (15–30 min), n (%)**			**<.01**
no	391 (86.9)	360 (80.0)	
yes	59 (13.1)	90 (20.0)	
**Oxytocin step 3 (> 30 min), n (%)**			**.03**
no	442 (98.2)	431 (95.8)	
yes	8 (1.8)	19 (4.2)	
**Never used oxytocin**	58 (12.9)	35 (7.8)	**0,01**

multiple doses of oxytocin were possible for each vignette

### Risk factors for delayed administration

Bivariate analysis in Vignette 1 indicated that the lack of promptness was significantly associated with age, years of experience, university hospital status, level of care, and volume of births per year (*p* < .05) in Vignette 1 (Table 4).

**Table 4:** Association between delay and individual and organizational characteristics in Vignette 1 (*N* = 450)

Characteristics	Delay > 15 min		***p***-value
	no, *N* = 353	yes, *N* = 97	
***Individual characteristics***			
Gender, n (%)			.3
Men	17 (68.0)	8 (32.0)	
Women	336 (79.1)	89 (20.9)	
Age, year, mean (SD)	34.19 (8.18)	36.67 (9.09)	**.02**
Experience, year, (SD)	10.84 (8.43)	13.36 (9.43)	**.02**
Full-time job, n (%)	267 (80.2)	66 (19.8)	.13
***Organizational characteristics***			
Maternity unit status, University, n (%)	90 (88.2)	12 (11.8)	**.01**
Level of care, n (%)			**.01**
level 1	89 (69.5)	39 (30.5)	
level 2	158 (80.2)	39 (19.8)	
level 3	106 (84.8)	19 (15.2)	
Volume of births per year, n (%)			**.01**
< 1500	129 (70.9)	53 (29.1)	
1500–2500	103 (85.1)	18 (14.9)	
> 2500	121 (82.3)	26 (17.7)	

We found no statistically significant association between promptness and midwives' characteristics in Vignette 2 (Table 5).

**Table 5:** Association between delay > 15 min and individual and organizational characteristics for Vignette 2 (*N* = 450)

Characteristics	Delay > 15 min		***p***-value
	no, *N* = 388	yes, *N* = 62	
***Individual characteristics***			
Gender, n (%)			.4
Men	20 (80.0)	5 (20.0)	
Women	368 (86.6)	57 (13.4)	
Age, years, mean (SD)	34.47 (8.36)	36.34 (8.78)	.09
Experience, year, mean (SD)	11.17 (8.64)	12.73 (9.08)	.2
Full time job, n (%)	289 (86.8)	44 (13.2)	.6
***Organizational characteristics***			
Maternity unit status, University n (%)	86 (84.3)	16 (15.7)	.5
Level of care, n (%)			.07
level 1	105 (82.0)	23 (18.0)	
level 2	178 (90.4)	19 (9.6)	
level 3	105 (84.0)	20 (16.0)	
Volume of births per year, n (%)			> 0.9
< 1500	157 (86.3)	25 (13.7)	
1500–2500	105 (86.8)	16 (13.2)	
> 2500	126 (85.7)	21 (14.3)	

Table 6 shows the odds ratios (OR) for the univariate and multivariable analysis for delay in Vignette 1. Age and level of care were not included because these were highly correlated with experience (Pearson’s *r* = 0.97) and volume of births (Pearson’s *r* = 0.74), respectively. Midwives were less likely to delay oxytocin administration when they worked in university hospital maternity units (ORa 0.47, 95% CI 0.21, 0.97, *p* = 0.049), or maternity units with between 1500-2500 births per year (ORa 0.49, 95% CI 0.26, 0.90, *p* = .024). Delay increased with years of experience (for each 10 years) (ORa 1.30, 95% CI 1.01, 1.69, *p* = 0.046).

**Table 6:** Univariate and multivariable analysis of delay > 15 min and individual and organizational characteristics for Vignette 1

Characteristics	Univariate			Multivariate		
	OR^a^	95% CI^a^	*p*-value	ORa^b^	95% CI	*p*-value
***Individual characteristics***						
Years of experience (for each 10 years)	1.37	1.07, 1.76	**.01**	1.30	1.01, 1.69	**.046**
Full-time job						
Yes [ref]	–			–		
No	1.46	0.88, 2.37	.13	1.43	0.85, 2.36	.2
***Organizational characteristics***						
Maternity units status						
Non-university [ref]	–			–		
University	0.41	0.21, 0.76	**.01**	0.47	0.21, 0.97	**.049**
Volume of births (per year)						
< 1500 [ref]	–			–		
1500–2500	0.43	0.23, 0.76	**.01**	0.49	0.26, 0.90	**.02**
> 2500	0.52	0.30, 0.88	**.02**	0.81	0.43, 1.51	.5

^a^*OR* Odds ratio, *CI* Confidence interval

^b^*ORa* Odds ratio adjusted for all characteristics

Since the frequency of errors was lower (62/450) and nothing was significant in the univariate analysis, Vignette 2 could not be evaluated for risk factors in a regression model.

## Discussion

### Main findings

Our study showed variations in the promptness of oxytocin administration for first-line treatment of PPH between the two different PPH situations.

We observed a delay in the administration of oxytocin treatment in both vignettes. The rate of inadequate responses corresponding to delay in oxytocin administration is high in vignette 2 (13.8%) and even higher for vignette 1 (21.6%). Similarly, the total absence of oxytocin use was also higher in vignette 1 (12.9%) than in vignette 2 (7.8%). For Vignette 1, a lower risk of lack of promptness in oxytocin administration was statistically associated with university hospital status and with maternity units with 1500–2500 births per year. Delay increased with years of experience (per 10-year interval).

### Clinical meaning

This difference in results between the two vignettes may be explained by the different clinical form of PPH in each. The more prompt pharmacological management in Vignette 2 than in Vignette 1 suggests that midwives’ management differed according to the clinical form of PPH. Although members of our team have previously showed strict adherence to all 14 of the guideline-based criteria rather than focusing on the timing of oxytocin administration.

It seems likely that the midwives started the pharmacological treatment more quickly in Vignette 2 because it was the easiest to implement in that situation, as the placenta had been delivered and the time since delivery — and without analgesia — was much longer [28]. It is technically faster to administer oxytocin to a patient without analgesia than to perform intrauterine gestures that require it. In step 1 for Vignette 1, it was expected that the midwife would both remove the placenta manually and administer oxytocin. It can be assumed that some midwives began by only removing the placenta, thinking that this would be effective in stopping the PPH; they thus delayed the oxytocin injection to step 2. This could explain the greater rate of inappropriate oxytocin use in vignette 1. This point indicates the need to clarify future guidelines [26, 39]. A qualitative approach could have been useful to help us understand these variations better.

Nos résultats sont cohérents avec la littérature. Une étude de cohorte de population (Pithagore6) a montré que l'ocytocine était administrée tardivement (> 10 min) ou pas du tout à 24,5 % des femmes atteintes d'HPP, un taux similaire à celui de 21,6 % dans Vignette 1. Les soins différés, par rapport à la prise en charge recommandée, ont été associés à un risque plus élevé d'HPP sévère, qui était 1,4 fois plus élevé chez les femmes qui ont reçu de l'ocytocine entre 10 et 20 min après le diagnostic d'HPP, et 1,9 fois plus élevé lorsqu'ils ont été administrés plus de 20 min après le diagnostic, [25]. Notre étude a montré l'administration d'ocytocine 30 min après le diagnostic dans 1,8 à 4,8 % des réponses, selon la vignette. Ceci est clairement considéré comme des soins inadéquats. Les lignes directrices indiquent la nécessité d'un agent utérotonique de deuxième intention dans les 30 minutes suivant le diagnostic d'HPP si le saignement persiste [12–16].

In the Netherlands, where the guideline for the timing of uterotonic medication is based on steps according to the current quantity of blood loss (and a uterotonic should be administered as soon as blood loss exceeds 500 mL), a prospective observational multicenter study found that this protocol was not followed in more than half of the cases for PPH > 500 mL despite systematic prophylactic administration of oxytocin as part of active management of the third stage of labor [40].

Various factors, including but not limited to delay, have previously been associated with the severity of PPH. Driessen showed that the risk of severe PPH in France was 1.5 times higher for PPH in non-teaching public compared with university hospitals (which are all public) [25]. Woisky’s results were similar in Dutch hospitals. Among potential determinants of adequate care, university hospital status has most often associated with better adherence to guidelines [40].

The association between facility birth volume and substandard care is interesting. Bouvier Colle et al. showed that inadequate care was nearly five times more common for deliveries in maternity facilities handling fewer than 500 births per year (only 3.3 times more frequent for substandard care, that is, inadequate and mixed, versus appropriate). Eight criteria — mainly involving timely clinical action — were defined to judge the quality of care based on the international literature or because the expert group considered them to be essential. When all the criteria were met, the cases were classified in the ‘appropriate’ category; both mixed and insufficient care were considered substandard [41]. In our study, we found that a birth volume of more than 1500 and fewer than 2500 births per year protected against this lack of promptness (ORa 0.49, 95% CI 0.26, 0.90, *p* = 0.024). Other studies suggest an increased risk of PPH in small units [42–44]. Snowden et al. also found that the rural hospitals with the lowest (4.5% for 50–599 births per year) and medium (3.3% for 600–1699 births per year) volumes had higher rates of PPH than higher-volume rural hospitals (1.7% for > 1700 births per year) [45].

Another important issue is the impact of years of experience on teaching skills and retention. In general, greater physician experience has been found to be negatively associated with medical knowledge, compliance with practice standards, and clinical outcomes [46]. In our study, years of experience was a risk factor for these delays (ORa 1.30, 95%CI 1.01, 1.69, *p* = 0.046). Moreover, we found similar results regarding advanced age and obstetricians’ application of the guidelines for the prevention of preterm birth [47]. Nevertheless, literature results in this domain remain disparate [48]. Maintaining a high level of competence through continuous training of health professionals is a major challenge that must be met to ensure a high level of care.

Marshall et al. showed in a multicenter longitudinal intervention study in Oregon (USA) that regular team training based on simulation decreased delays in oxytocin administration in non-academic centers. The team initiated the use of oxytocin 48 s earlier (SD 66, *p* = 0.003) compared with their initial performance [49]. In a prospective observational study at an academic medical center, Dillon et al. reported a significant reduction in the variation in time between uterotonic drug administration and blood transfusion after implementing a simulation program (*p* = 0.035) [50]. Finally Nelissen et al. carried out a half-day obstetric simulation-based training in a rural referral hospital in Tanzania as part of a prospective intervention study and found that the proportion of women who received oxytocin as part of PPH management increased significantly, from 43.0% before training to 61.2% afterwards (*p* = 0.04) [51].

### Strengths and limitation

One strength is that the characteristics of the participating units were similar to those of French maternity units overall [52]. Moreover this study investigated the issue of French midwives’ adherence to current guidelines for first-line treatment of PPH by using clinical vignettes. This methodology is a less difficult way to assess midwifery skills in emergency situations, more feasible than RCTs in these stressful conditions, with less complex recruitment and logistics. The use of vignettes may be considered a lower level of evidence than a cohort study, but it nonetheless remains an inexpensive and effective way to assess clinical practice in a more detailed (dose, route, timing) and individualized (i.e., related to caregiver) manner than is possible with medical records [53]. Moreover, the results of population-based studies are similar to ours and suggest their good external validity. Clinical vignettes put professionals in a situation that allows them to exercise their reasoning and clinical approach in a context close to their actual practice conditions [36, 38]. They appear to be reliable for assessing drug prescription practices [37] and enable the evaluation of elements that are less well traced in medical records. Woisky was able to show through video recordings that in general the actual care given was quite substantially underreported in medical records [40].

This study also has limitations. An indirect approach to the emergency context by clinical vignette may result in social desirability bias that can lead to an overestimation of appropriate management and compliance. Nevertheless, our results show variations in practice and inappropriate practices, which are therefore likely to be underestimated. Moreover, we cannot rule out a selection bias linked to the voluntary participation of midwives, leading to a response rate of 41%. It is likely that only those midwives most interested in the topic responded to the survey. Another limitation may be the international generalizability of our results. Although it can be assumed that the management of PPH differs from country to country, this management is still based on guidelines requiring that oxytocin be administered promptly after diagnosis. Furthermore, we chose to focus on the administration of oxytocin as treatment because it is the first-line uterotonic that can be administered autonomously by French midwives. While we did not take second-line uterotonics into account because they must be prescribed by an obstetrician, it was not considered an error to switch directly to a second-line uterotonic. Lastly, this study involved only midwives and the results cannot be generalized to other practitioners. However, as noted above, midwives play a key role in the diagnosis and initial management of PPH.

## Conclusions

In conclusion, this multicenter cross-sectional study using clinical vignettes showed delays in oxytocin administration for first-line treatment of PPH. Because delay in treatment is a major cause of preventable maternal morbidity in PPH, these findings suggest that continuing training of midwives should be considered; especially in small maternity units.

## Supplementary Information

**Additional file 1.**

**Additional file 2.**

## Data Availability

All relevant data are within the paper and its Supporting Information files.

## Abbreviations

PPH: Postpartum hemorrhage
RCT: Randomized controlled trial
WHO: World Health Organization
