# Supplementary material for: Promptness of oxytocin administration for first-line treatment of postpartum hemorrhage: a national vignette-based study among midwives
Source: BMC Pregnancy Childbirth. 2022 Apr 23;22:353. doi: 10.1186/s12884-022-04648-5 (PMC9034651; doi:10.1186/s12884-022-04648-5)
Supplement: Supplementary file 1 — Additional file 1. [file 12884_2022_4648_MOESM1_ESM.pdf]

Vignette 1

**Age** : 33years	**Antecedent : cesarean section 3 years earlier**
**Gravida** 2	**Pregnancy** : streptococcus B carriage
**Origin** : french	**Term** : 38 weeks of gestation
**BMI** : 20	**Spontaneous labor**

Pre-operative blood test : haemoglobin: 10.2g/dL, platelet: 235G/L

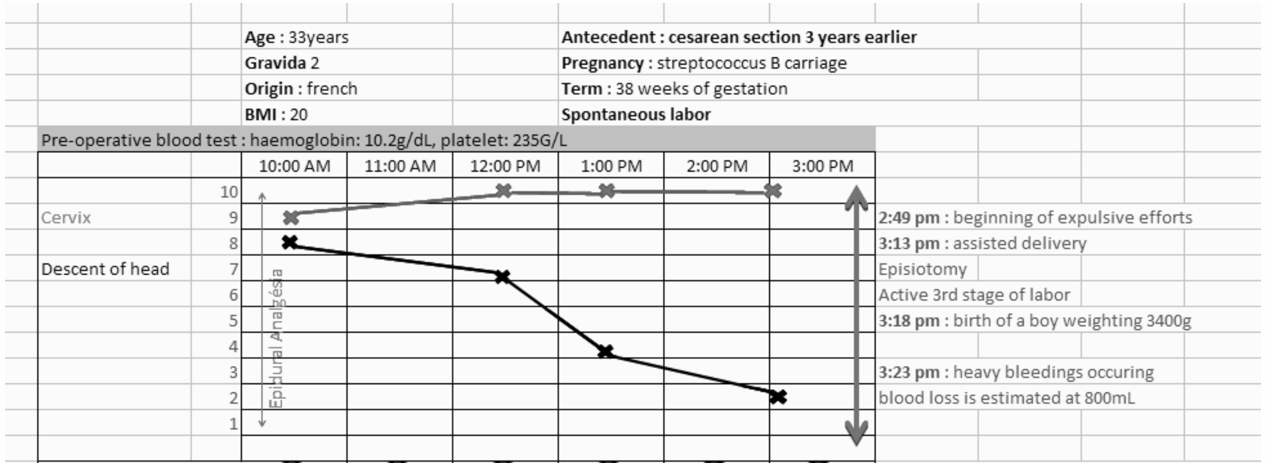

2:49 pm : beginning of expulsive efforts
3:13 pm : assisted delivery
Episiotomy
Active 3rd stage of labor
3:18 pm : birth of a boy weighting 3400g

3:23 pm : heavy bleedings occuring
blood loss is estimated at 800mL

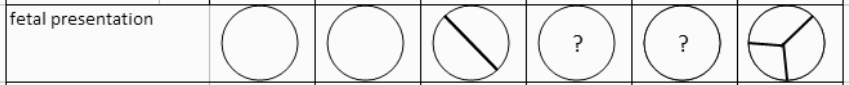

		10:00 AM	11:00 AM	12:00 PM	1:00 PM	2:00 PM	3:00 PM
ruptured membranes		Intact		Rupture of membranes	clear amniotic fluid	clear amniotic fluid	clear amniotic fluid
fetal heart rate (FHR)		140bpm	140bpm	140bpm	140bpm	140bpm	140bpm
Frequency of Contractions		3-4/10min	3-4/10min	3-4/10min	3-4/10min	3-4/10min	
Constant	BP<br>temp	14/7	11/7	11/7<br>37,4	11/7	11/7	11/7<br>37,7
Behavior		Algesic	Calm	Calm	Calm	Calm	Calm
Treatment	Ringer Lactate	Δ<br>antibiotic			Bladder catheterization	Δ<br>antibiotic	Bladder catheterization

What measures would you perform **within the next 15 minutes** ?

Vignette 1

Despite your actions, bleeding persists. Uterus is hypotonic

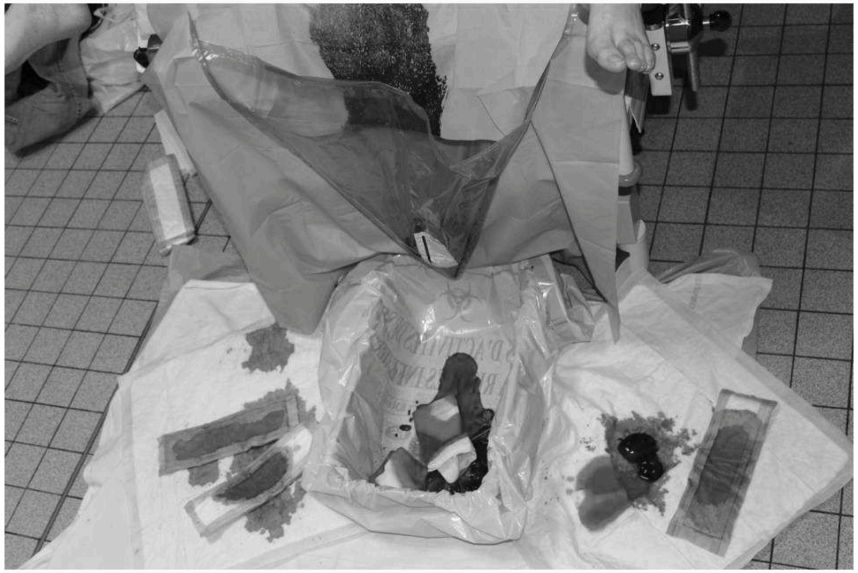

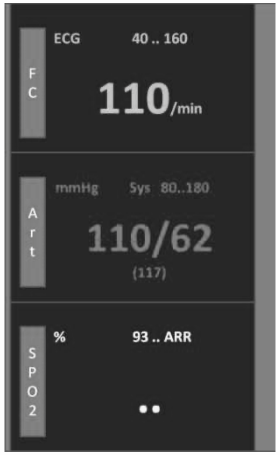

What measures would you perform **within the next 15 minutes** ?

Vignette 1

30 minutes later, despite your actions, bleedings persists. The uterus is hypotonic when you stop the uterine massage. The patient does not feel very well and is dizzy.

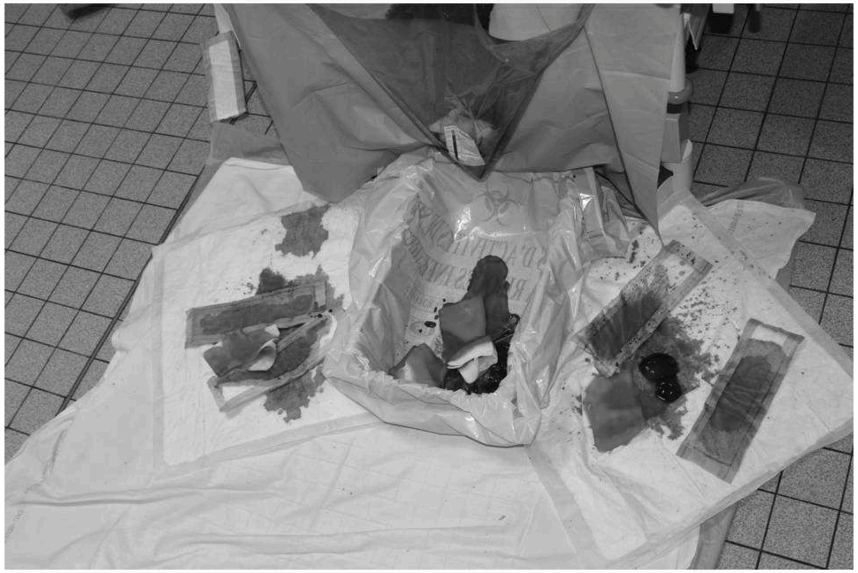

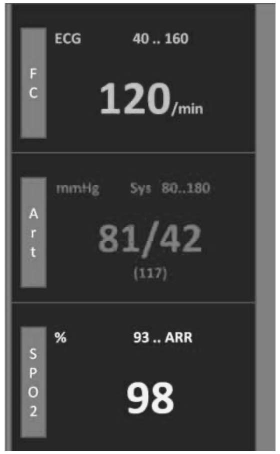

What measures you propose at this stage ?
