# Supplementary material for: Promptness of oxytocin administration for first-line treatment of postpartum hemorrhage: a national vignette-based study among midwives
Source: BMC Pregnancy Childbirth. 2022 Apr 23;22:353. doi: 10.1186/s12884-022-04648-5 (PMC9034651; doi:10.1186/s12884-022-04648-5)
Supplement: Supplementary file 2 — Additional file 2. [file 12884_2022_4648_MOESM2_ESM.pdf]

Vignette 2

Age : 31years

Gravida 1

Origin : Moroccan

BMI : 23

Course of pregnancy uneventful

Term : 40 weeks of gestation

Spontaneous labor

Pre-operative blood test : haemoglobin: 13.1g/dL, platelet: 178G/L

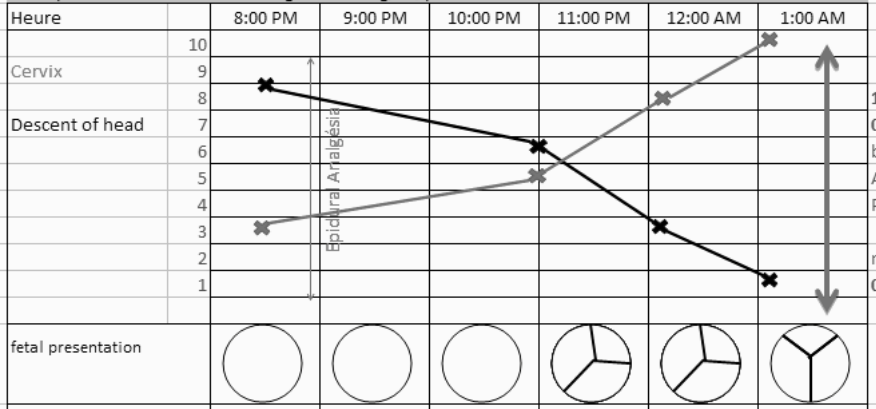

12:40 am: beginning of expulsive efforts
01:05 am : spontaneous vaginal delivery
boy weighting 3640g
Active 3rd stage of labor
Placenta delivered

minimal bleeding appears and stops with uterus massage
02:30 am : minimal bleeding persists, blood loss is estimated at 650mL

Heure		8:00 PM	9:00 PM	10:00 PM	11:00 PM	12:00 AM	1:00 AM
ruptured membranes			Rupture of membranes	clear amniotic fluid	clear amniotic fluid	clear amniotic fluid	brown amniotic fluid
fetal heart rate (FHR)		140bpm	140bpm	140bpm	140bpm	160bpm	160bpm
Frequency of Contractions		4/10min	3-4/10min	3-4/10min	3-4/10min	4/10min	4/10min
Constant	BP	12/7	12/7	11/7	11/7	11/7	
	temp	36,9					
Behavior		Algic	Algic	Calm	Calm	Calm	Calm
Treatment	Ringer Lactate				Oxytocine		Bladder catheterization

What measures would you perform **within the next 15 minutes** ?

Vignette 2

Despite your actions, bleeding persists in thin stream. The uterus is tonic.

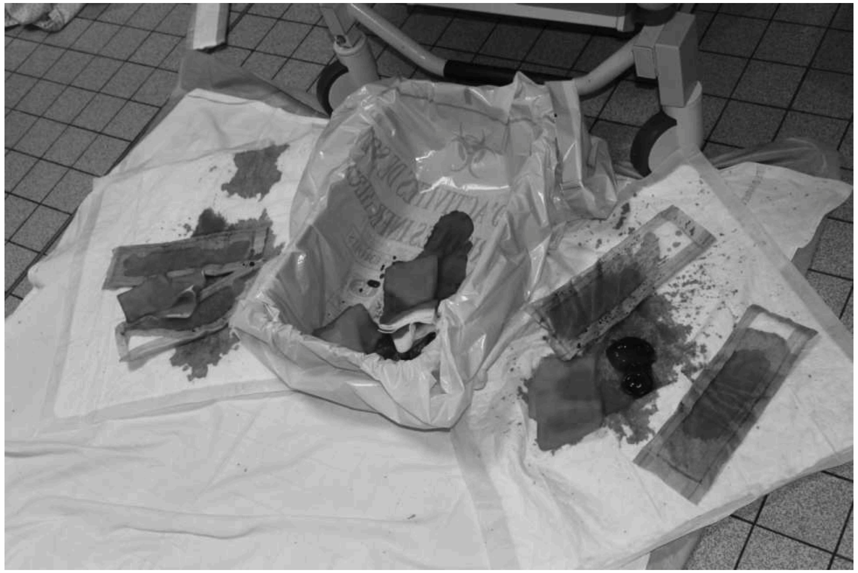

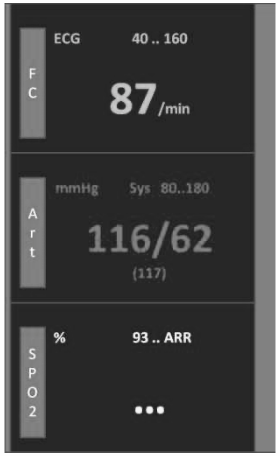

What measures would you perform **within the next 15 minutes** ?

Vignette 2

30 minutes later, despite your actions, bleeding is more abundant. The uterus is tonic.

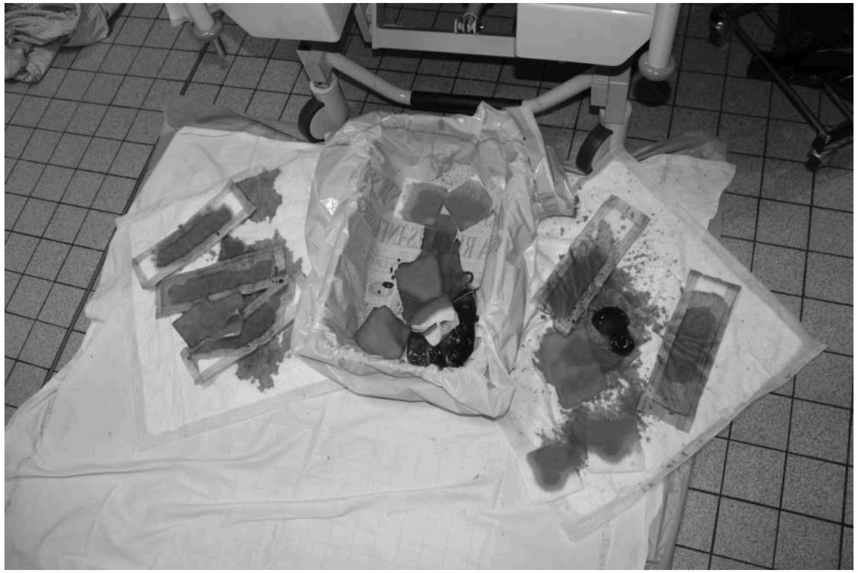

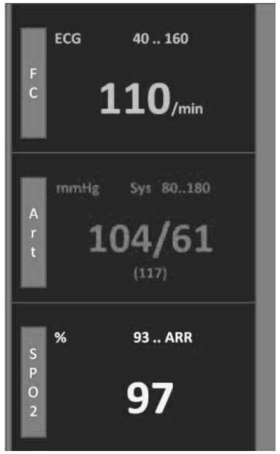

What measures you propose at this stage ?
